# 3D echocardiography, arterial stiffness, and biomarkers in early diagnosis and prediction of CHOP-induced cardiotoxicity in non-Hodgkin’s lymphoma

**DOI:** 10.1038/s41598-020-75043-3

**Published:** 2020-10-28

**Authors:** Diana Mihalcea, Maria Florescu, Ramona Bruja, Natalia Patrascu, Ana-Maria Vladareanu, Dragos Vinereanu

**Affiliations:** 1 University of Medicine and Pharmacy Carol Davila, Bucharest, Romania; 2 University and Emergency Hospital, Bucharest, Romania

**Keywords:** Chemotherapy, Cardiomyopathies, Cardiology, Oncology

## Abstract

CHOP (cyclophosphamide, doxorubicin, vincristine, prednisone) represents standard chemotherapy in non-Hodgkin's lymphoma (NHL) with risk of cardiotoxicity. To define new parameters, such as 3D myocardial deformation, arterial stiffness, and biomarkers for early diagnosis and prediction of cardiotoxicity. 110 NHL patients with LVEF > 50%, scheduled for CHOP, were evaluated at baseline, after third cycle and chemotherapy completion. 3DE assessed LVEF and myocardial deformation: longitudinal (LS), radial, circumferential, area strain. Echo-tracking analysed arterial stiffness: PWV, β index, wave intensity. Troponin I and NT-pro-BNP were measured. After chemotherapy completion, 18 patients (16%) (group I) developed cardiotoxicity (LVEF decrease < 50%, with > 10% from baseline); 92 patients (group II) did not. Significant reduction of 3D LV deformation and increase of arterial stiffness developed starting with third cycle, with greater changes in group I. LS reduction and PWV increase after third cycle were the best independent predictors for LVEF decrease; the association of LS decrease by > 19% and PWV increase by > 27% after third cycle predicted cardiotoxicity after chemotherapy completion (90% sensitivity and 81% specificity). 3D LS and PWV can detect early chemotherapy-induced cardiotoxicity and predict LVEF decline. These parameters should be incorporated in clinical protocols to monitor cardiovascular function during chemotherapy and early intervention.

## Introduction

Non-Hodgkin’s lymphoma (NHL), the most common hematological neoplasia, represents an important public health problem, due to increased risk of morbidity and mortality^1^. CHOP regimen (cyclophosphamide, doxorubicin, vincristine, prednisone) is the standard chemotherapy for NHL, providing high rate of cure and reduced recurrence of disease, with good tolerability^1^. The most severe side effect of these agents, which might determine early withdrawal of therapy, is represented by cardiotoxicity^2^. Cardiac dysfunction, mainly due to anthracyclines, may be present from asymptomatic forms, with reduction of left ventricular ejection fraction (LVEF), in 57% of patients, to overt heart failure in 16% of patients^2–4^. Cardiac dysfunction induced by chemotherapy is defined by the current guidelines as a LVEF decrease below 50%, with more than 10 percentage points, 2 to 3 weeks after initiation of therapy^2^. Three-dimensional echocardiography (3DE) is the method recommended for assessing LVEF^2^. However, 3D LVEF assessment allows only late diagnosis of cardiotoxicity, often irreversible. Thus, description of new and simple parameters of myocardial deformation and arterial stiffness, affected before decrease of LVEF, might diagnose subclinical, early changes of cardiac function induced by CHOP therapy, and might be able to predict cardiotoxicity^5,6^. Moreover, a role in the early diagnosis of cardiotoxicity can be added by the cardiac biomarkers. Thus, troponin I (TnI), marker of myocardial injury, may increase before occurring of symptomatic cardiotoxicity, while NT-pro-BNP, marker of increased preload, can predict onset of LV dysfunction^7^.

Our aim was to define new parameters, such as 3D myocardial deformation, arterial stiffness, and biomarkers, in early detection and prediction of cardiovascular toxicity, in NHL patients receiving CHOP chemotherapy.

## Results

### Baseline characteristics

110 patients were included in the study: 18 patients (16%) developed cardiotoxicity at the end of CHOP chemotherapy (group I), whereas 92 subjects (84%) did not. Baseline characteristics of two groups are presented in Table 1. All patients completed the whole chemotherapeutic treatment without any cardiovascular complications.

**Table 1:** Baseline characteristics of patients from study group, group I (with cardiotoxicity), and group II (without cardiotoxicity).

Characteristics	Study group (n = 110)	Group I (n = 18)	Group II (n = 92)	p-value
Age (years)	58 ± 11	56 ± 14	59 ± 6	0.72
Male (n, %)	51 (46)	8 (44)	43 (46)	0.78
Body mass index (kg/m^2^)	25 ± 3	26 ± 2	24 ± 2	0.56
Systolic BP (mmHg)	123 ± 15	128 ± 12	122 ± 16	0.83
Diastolic BP (mmHg)	74 ± 10	76 ± 8	73 ± 10	0.76
Heart rate (beats/min)	75 ± 11	80 ± 11	73 ± 11	0.07
**Cardiovascular risk factors:**				
Hypertension (n, %)	19 (17)	4 (22)	15 (16)	0.55
Smoking (n, %)	10 (9)	2 (11)	8 (8)	0.82
Diabetes mellitus (n, %)	5 (4)	2 (11)	3 (3)	0.85
Dyslipidemia (n, %)	9 (8)	3 (16)	6 (6)	0.78
**Stage of NHL**				
1 (n, %)	9 (8)	1 (5)	8 (9)	0.93
2 (n, %)	16 (15)	2 (12)	14 (15)	0.81
3 (n, %)	53 (48)	8 (44)	45 (49)	0.88
4 (n, %)	32 (29)	7 (39)	25 (27)	0.06
Cumulative dose of Doxorubicin (mg)	429 ± 61	443 ± 45	421 ± 56	0.10

BP = blood pressure; NHL = non-Hodgkin’s lymphoma; p-value between groups I and II; values are shown as mean ± SD or percentage.

### Echocardiographic parameters

**Figure 1:**
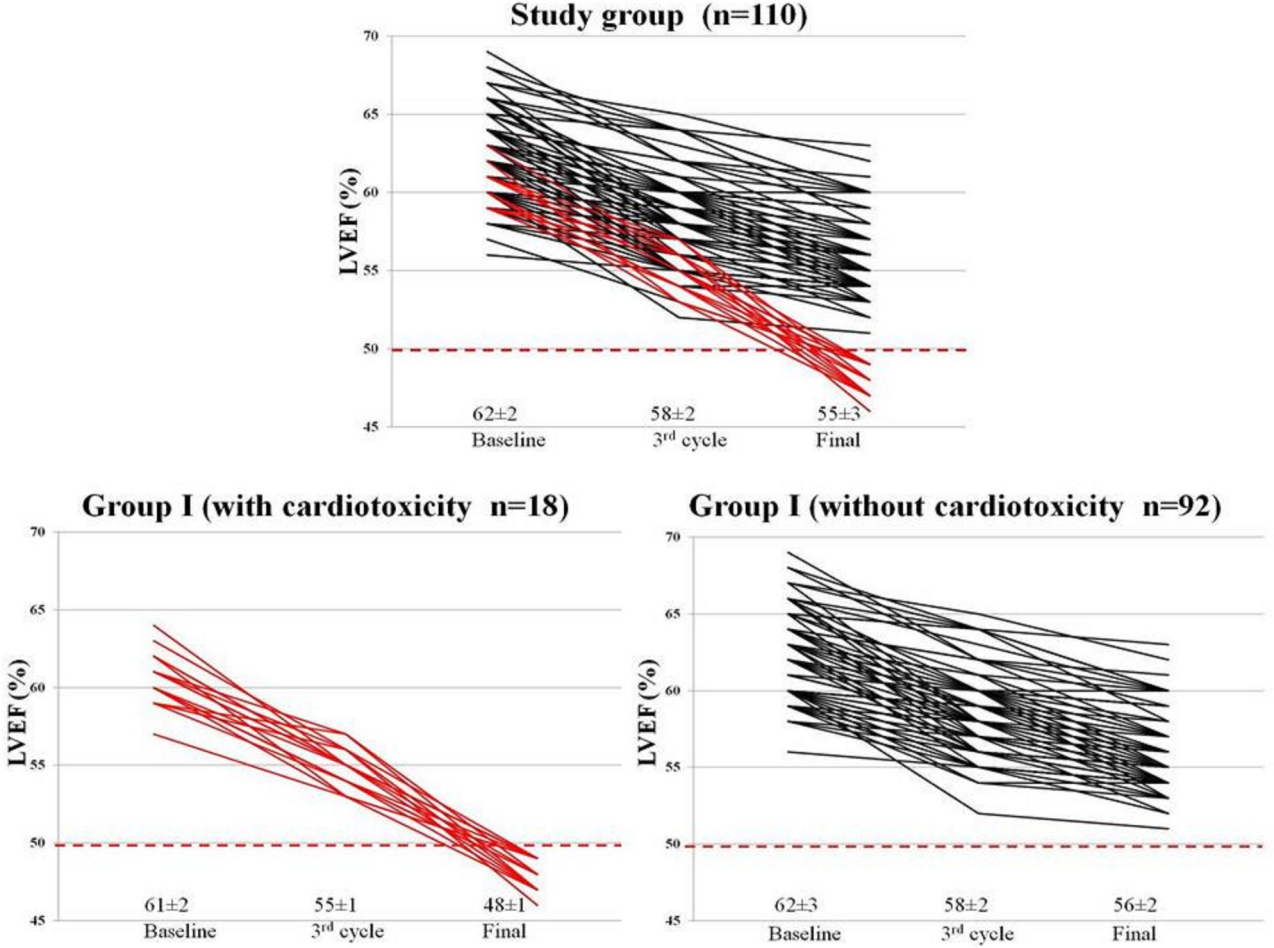
Individual values of 3D left ventricular ejection fraction (LVEF) at baseline, after the third cycle, and after CHOP completion chemotherapy in study group, group I (with cardiotoxicity), and group II (without cardiotoxicity).

**Figure 2:**
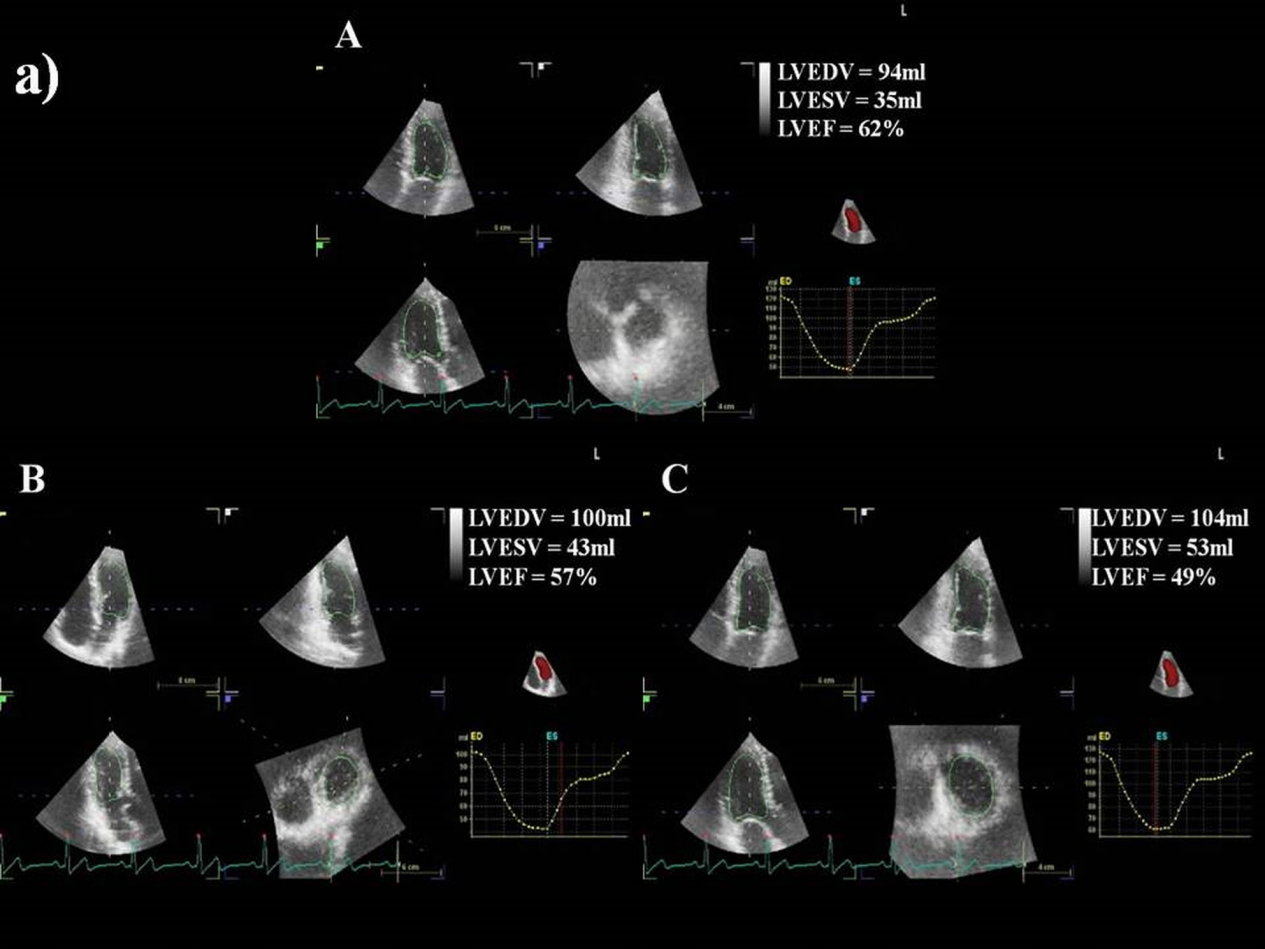

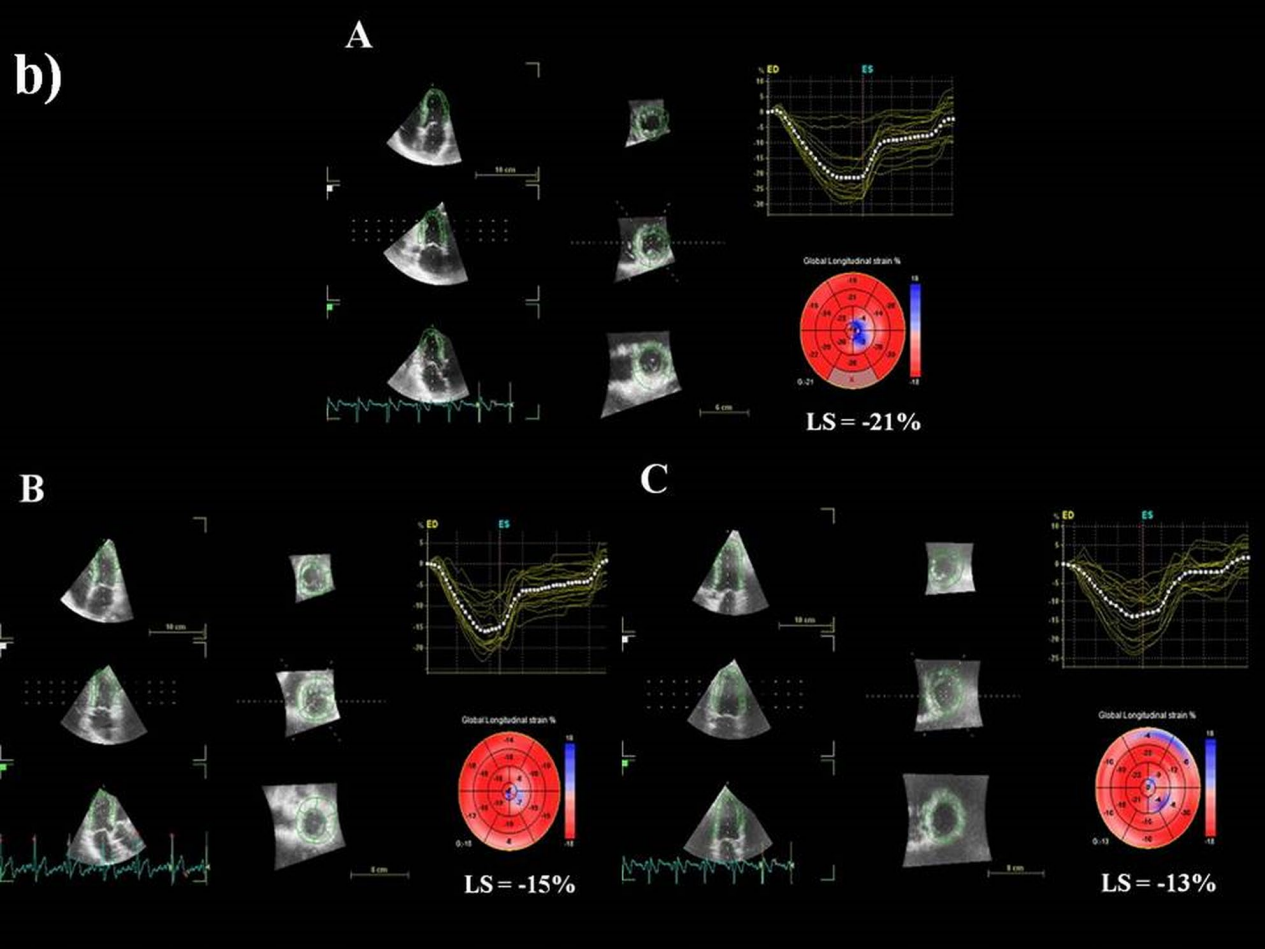
(a) An example of reduction of 3D left ventricular ejection fraction (LVEF) from baseline **(A)** to third cycle **(B),** and after completion of CHOP therapy **(C)**, in a patient who developed cardiotoxicity (from group I). b) Reduction of 3D longitudinal strain (LS) from baseline **(A)** to third cycle **(B)**, and after completion of CHOP therapy **(C)**, in the same patient who developed cardiotoxicity (from group I).

3D echocardiographic parameters at baseline, after third cycle, and at the end of CHOP chemotherapy are shown in Table 2. Baseline 3D LVEF was 62 ± 2%, with no differences between groups; after third cycle of chemotherapy, there was a decrease of 3D LVEF in the study group, with significant difference between the two groups, which persisted after treatment completion (Fig. 1). 3D myocardial deformation parameters were similar at baseline between groups. After third cycle of chemotherapy, there was a significant decrease of LS, CS, RS, and AS in the study group, persistent after final cycle of therapy, with more important reduction in group I (Table 2). Examples of 3D LVEF reduction (a) and 3D LS (b) after third cycle, and at the end of therapy, by comparison with baseline, in a patient from group I, who developed cardiotoxicity, are presented in Fig. 2.

**Table 2:** 3D echocardiographic and arterial stiffness parameters, and cardiac biomarkers of patients from study group, group I (with cardiotoxicity), and group II (without cardiotoxicity).

Parameters	CHOP chemotherapy	Study group (n = 110)	Group I (n = 18)	Group II (n = 92)	p-value†
LVEDV (ml)	Baseline	91 ± 19	96 ± 18	90 ± 20	0.65
	3rd cycle	94 ± 16	100 ± 16	92 ± 14	0.22
	Final	100 ± 19	107 ± 15*	97 ± 20	0.01
LVESV (ml)	Baseline	31 ± 7	35 ± 6	33 ± 8	0.43
	3rd cycle	37 ± 8	41 ± 7	36 ± 8	0.06
	Final	43 ± 9*	45 ± 8*	42 ± 9*	0.01
LVEF (%)	Baseline	62 ± 2	61 ± 2	62 ± 3	0.40
	3rd cycle	58 ± 2*	55 ± 1*	58 ± 2*	0.003
	Final	55 ± 3*	48 ± 1*	56 ± 2*56 ± 2*	0.0001
LS (-%)	Baseline	22.6 ± 1.4	22.7 ± 1.2	22.6 ± 1.5	0.71
	3rd cycle	18.2 ± 2.7*	14.8 ± 1.8*	19.7 ± 1.8*	0.0001
	Final	15.6 ± 3.2*	11.5 ± 1.2*	16.5 ± 2.1*	0.0001
CS (-%)	Baseline	22.3 ± 1.5	22.2 ± 1.0	22.3 ± 1.6	0.83
	3rd cycle	18.3 ± 2.2*	15.0 ± 1.4*	19.3 ± 1.2*	0.001
	Final	15.6 ± 2.6*	11.9 ± 1.1*	16.2 ± 1.5*	0.001
RS (%)	Baseline	61.1 ± 4.8	61.3 ± 5.1	61.0 ± 4.7	0.81
	3rd cycle	53.1 ± 5.7	48.6 ± 6.8	54.5 ± 4.6	0.001
	Final	47.0 ± 6.5*	44.7 ± 8.2*	50.7 ± 4.9*	0.01
AS (-%)	Baseline	43.4 ± 2.9	42.8 ± 2.6	44.1 ± 1.9	0.61
	3rd cycle	37.5 ± 3.6	37.4 ± 2.7*	38.3 ± 2.2	0.39
	Final	33.6 ± 3.5*	31.8 ± 3.2*	35.3 ± 1.7*	0.01
PWV (m/sec)	Baseline	6.7 ± 1.1	6.4 ± 1.1	6.8 ± 1.1	0.17
	3rd cycle	7.2 ± 1.2*	7.4 ± 1.4*	7.1 ± 1.1	0.05
	Final	7.8 ± 1.5*	8.9 ± 1.6*	7.4 ± 1.3*	0.002
AIX (%)	Baseline	19.6 ± 6.6	15.9 ± 5.2	20.7 ± 6.7	0.20
	3rd cycle	26.1 ± 7.9*	29.6 ± 7.8*	25.0 ± 7.7	0.06
	Final	32.5 ± 10.2*	39.7 ± 7.9*	29.4 ± 8.7*	0.01
β index	Baseline	9.4 ± 2.7	7.5 ± 1.9	10.0 ± 2.6	0.3
	3rd cycle	12.4 ± 5.0*	14.8 ± 8.5*	11.7 ± 3.1*	0.02
	Final	15.2 ± 7.3*	20.7 ± 12.0*	13.5 ± 4.0*	0.001
Ep (kPa)	Baseline	82.1 ± 16.8	85.2 ± 13.7	80.7 ± 17.2	0.06
	3rd cycle	94.2 ± 22.3*	101.5 ± 15.9*	85.6 ± 14.9	0.02
	Final	107.4 ± 19.8*	110.3 ± 18.6*	104.8 ± 18.5*	0.03
AC (mm^2^/kPa)	Baseline	0.86 ± 0.1	0.84 ± 0.2	0.86 ± 0.2	0.07
	3rd cycle	0.99 ± 0.2*	1.2 ± 0.1*	0.9 ± 0.3	0.01
	Final	1.2 ± 0.18*	1.3 ± 0.2*1.3 ± 0.2*	1.1 ± 0.4*	0.006
WI (m/sec)	Baseline	5.5 ± 1.3	5.6 ± 1.4	5.0 ± 1.8	0.24
	3rd cycle	6.4 ± 1.7*	7.2 ± 1.5*	6.2 ± 1.7	0.03
	Final	8.5 ± 1.9*	8.8 ± 1.5*	7.8 ± 1.5*	0.001
Negative area (mmHg x m/s^2^)	Baseline	82.3 ± 2.6	84.3 ± 3.5	81.2 ± 2.7	0.08
	3rd cycle	89.4 ± 3.0*	92.5 ± 2,6*	87.4 ± 3.3	0.05
	Final	95.2 ± 3.4*	97.1 ± 3.1*	92.6 ± 2.8*	0.04
Tn I (ng/ml)	Baseline	0.018 ± 0.005	0.007 ± 0.002	0.003 ± 0.001	0.07
	3rd cycle	0.036 ± 0.005*	0.045 ± 0.005	0.021 ± 0.005	0.09
	Final	0.058 ± 0.017*	0.078 ± 0.006*	0.025 ± 0.004	0.004
NT-pro-BNP (pg/ml)	Baseline	67 ± 21	74 ± 15	58 ± 14	0.66
	3rd cycle	89 ± 20	94 ± 17	83 ± 15	0.50
	Final	102 ± 26	110 ± 14	98 ± 23	0.19

*AC* arterial compliance; *AIX* augmentation index; *AS* area strain; *CS* circumferential strain; *Ep* Young modulus of stiffness; *LS* longitudinal strain; *LV* left ventricle; *LVEDV* left ventricular end-diastolic volume; *LVEF* left ventricular ejection fraction; *LVESV* left ventricle end-systolic volume; *NT-pro-BNP* N-terminal pro-brain natriuretic peptide; *PWV* pulse wave velocity; *RS* radial strain; *Tn I* troponin I; *WI* wave intensity.

*p < 0.05 within each group versus baseline.

^†^p-value between groups I and II; values are shown as mean ± SD.

Arterial stiffness parameters had no significant differences between the two groups at baseline. After third cycle, arterial stiffness increased, being persistently increased at the end of therapy, with higher values in the group with cardiotoxicity (Table 2).

### Biomarkers

TnI levels were increased after third cycle and persistent after CHOP completion treatment, with higher values in group I than in group II (Table 2). Although NT-pro-BNP levels increased numerically during chemotherapy, there was no significant difference between the two groups (Table 2).

### Prediction models

By univariate analysis, reduction of LVEF at the end of therapy correlated with reduction of myocardial deformation, increase of arterial stiffness, TnI, and doxorubicin dose (Table 3). By stepwise multivariable linear regression analysis, in a model that included variation of 3D myocardial deformation (LS, RS, AS), arterial stiffness (PWV, β index, WI), and doxorubicin dose, LS and PWV after the third cycle of CHOP therapy were the best independent predictors of 3D LVEF decrease after CHOP treatment (R^2^ = 0.516, p = 0.0001).

**Table 3:** Correlations (r values) between change of 3D LVEF and (A) 3D myocardial deformation parameters and CHOP final doses and (B) arterial stiffness parameters and cardiac biomarkers.

A	3D Myocardial deformation				CHOP chemotherapy				
3D LVEF	LS	CS	RS	AS	C	H	O	P	
	0.703*	0.433*	0.388*	0.571*	− 0.165	− 0.566*	− 0.188	− 0.112	

B	Arterial stiffness							Cardiac biomarkers	
3D LVEF	PWV		β index	Ep	AC	WI	NA	AIX	NT-pro-BNP
	− 0.637*	− 0.233	− 0.531*	− 0.340*	− 0.337*	− 0.584*	− 0.188	− 0.228*	− 0.182

*AC* arterial compliance; *AIX* augmentation index; *AS* area strain; *C* cyclophosphamide; *CS* circumferential strain; *Ep* Young modulus of stiffness; *H* hydroxydaunorubicin (doxorubicin); *LS* longitudinal strain; *LVEF* left ventricular ejection fraction; *NA* negative area; *NT-pro-BNP* N-terminal pro-brain natriuretic peptide; *O* oncovin (vincristine); *P* prednisone; *PWV* pulse wave velocity; *RS* radial strain; *Tn I* troponin I; *WI* wave intensity.

*p < 0.05.

ROC analysis showed that a decrease of 3D LS by more than 19% from baseline, after the third cycle, predicted the occurrence of cardiotoxicity at the end of CHOP chemotherapy with a sensitivity of 89% and a specificity of 85%; an increase of PWV by more than 27% after the third cycle comparing with baseline, predicted also the occurrence of cardiotoxicity at the end of chemotherapy with a sensitivity of 88% and a specificity of 83% (Table 4). More than that, by binary logistic regression and ROC curve, the association of 3D LS and PWV identified patients with cardiotoxicity at the end of treatment with a c-statistic (AUC) of 96% (Table 4).

**Table 4:** Sensitivity, specificity, positive predictive value, negative predictive value and accuracy of 3D myocardial deformation and arterial stiffness parameters to predict cardiotoxicity after CHOP therapy completion.

Parameter	Sensitivity (%)	Specificity (%)	PPV (%)	NPV (%)	AUC (95% CI)	p-value
Combination of LS decrease > 19% and PWV increase > 27%	90	81	87	85	0.965 (0.912–0.992)	0.0001
LS decrease > 19%	89	85	83	86	0.919 (0.848–0.983)	0.0001
AS decrease > 28%	88	83	76	78	0.857 (0.733–0.972)	0.0001
CS decrease > 37%	78	81	68	73	0.842 (0.716–0.964)	0.001
RS decrease > 43%	62	70	57	77	0.761 (0.618–0.904)	0.005
PWV increase > 27%	88	83	81	82	0.901 (0.833–0.967)	0.0001
β index increase > 34%	80	77	71	73	0.832 (0.713–0.933)	0.0002
WI increase > 39%	81	72	69	73	0.774 (0.592–0.881)	0.0002
Ep increase > 49%	60	66	54	71	0.723 (0.603–0.878)	0.005
AC increase > 46%	61	64	55	67	0.658 (0.483–0.710)	0.005

*AC* arterial compliance; *AUC* area under the curve; *AS* area strain; *CI* confidence interval; *CS* circumferential strain; *Ep* Young modulus of stiffness; *LS* longitudinal strain; *NPV* negative predictive value; *PPV* positive predictive value; *PWV* pulse wave velocity; *RS* radial strain; *WI* wave intensity.

Intraobserver, interobserver, and test–retest variabilities for 3D LV EF, volumes, and myocardial deformation parameters, are shown in Table 5, with a feasibility of > 90% for all myocardial segments.

**Table 5:** Intraobserver variability, interobserver variability, and repeatability of 3D LV EF, volumes, and myocardial deformation parameters.

Parameter	Intraobserver variability (%)	Interobserver variability (%)	Repeatability (%)
LVEF	± 4.39	± 4.64	± 4.62
LVEDV	± 5.38	± 5.97	± 5.83
LVESV	± 7.81	± 8.54	± 8.21
LS	± 6.14	± 7.25	± 8.46
AS	± 7.33	± 7.02	± 7.41
CS	± 8.20	± 9.40	± 9.12
RS	± 8.25	± 9.04	± 7.4

*AS* area strain; *CS* circumferential strain; *LS* longitudinal strain; *LVEDV* left ventricular end-diastolic volume; *LVEF* left ventricular ejection fraction; *LVESV* left ventricular end-systolic volume; *RS* radial strain.

## Discussion

In a study on 110 patients with NHL, we showed that the assessment of 3D myocardial deformation, arterial stiffness, and cardiac biomarkers can detect and predict subclinical cardiotoxicity. From our best knowledge, this is the first study assessing, in a comprehensive way, systolic LV function by EF and myocardial deformation using 3D echocardiography, vascular function by arterial stiffness, and cardiac biomarkers, in a large population diagnosed with NHL, who received a single CHOP-type chemotherapeutic regimen.

CHOP regimen, very effective in achieving complete remission of NHL, has numerous side effects that cause increased morbidity and mortality^1,2^. Cardiotoxicity remains the most feared side effect of chemotherapy, with growing incidence of 20–30%^2,8^. Myocardial direct toxic effect of chemotherapy can lead to irreversible cardiomyopathy, with diastolic and/or dysfunction, which progresses to heart failure^2–4^. Using myocardial deformation and cardiac biomarkers, in patients with NHL receiving CHOP therapy, we demonstrated that an early decrease of longitudinal strain with more than 19%, after third cycle of therapy, predicts occurrence of cardiotoxicity after CHOP completion treatment. These results are essential for monitoring patients during chemotherapy and for early detection of patients at risk for heart failure.

Echocardiography is the best method for monitoring patients before, during, and after chemotherapy, being widely available, noninvasive, and cost effective^4^. LVEF is the standard parameter used to diagnose cardiotoxicity^2^. Cardiotoxicity is defined as LVEF decrease with more than 10 percentage points, to a value below 50%, evaluated 2–3 weeks after initiation therapy^2^. To avoid LV geometric assumptions, foreshortening, and inappropriate visualization of apex, we measured LVEF by 3DE, as recommended by current guidelines^2^. Compared to 2D echocardiography, 3DE has better intra- and inter- observer variability, and test–retest variability, and allows a more accurate assessment of LV volumes and ejection fraction, with better agreement with cardiac magnetic resonance (CMR), considered the “gold standard”^2,9^. In cancer patients, 3D LVEF is more sensitive and robust in detecting anthracyclines-induced cardiotoxicity in patients with different forms of solid or hematological tumors^10,11^.

Of the four chemotherapeutic agents used in CHOP therapy (cyclophosphamide, doxorubicin, vincristine, and prednisone), doxorubicin is by far the most important for occurrence of heart injury^2^. Risk for cardiotoxicity is greater when cumulative dose of doxorubicin is high, in association with other anticancer drugs or radiotherapy, or in single injection administration (versus long infusion)^2,3^. A dose of doxorubicin that exceeds 500 mg/m^2^ is an important factor in occurrence of cardiotoxicity, while doses below 300 mg/m^2^ are associated with low risk^2^. Our study showed a significant reduction of LVEF after a moderate dose of doxorubicin (429 ± 61 mg); however, asymptomatic cardiac dysfunction occurred since third cycle of CHOP, after a low dose of anthracycline (183 ± 42 mg), with reduction of all 3D myocardial deformation parameters. Our findings are consistent to those of Olivieri et al.^12^, Boyd et al.^13^, and Yu et al.^14^, who demonstrated occurrence of cardiotoxicity in 11–35% of patients, with frequent subclinical forms, at low and moderate doses of doxorubicin, between 150 and 450 mg/m^2^. Cyclophosphamide can induce cardiotoxicity in less than 2% of cases, manifested by arrhythmias and arterial or venous thromboembolism, but the dose used in CHOP regimen is too low to induce cardiac dysfunction by itself^2,15^; in fact, none of our patients experienced arrhythmia or thromboembolic events. Vincristine can lead to different non-cardiac side effects, but no cardiac side effects have been reported^16^. Corticosteroids may induce hypertension or atrial fibrillation^2^, but none of our patients developed atrial fibrillation, while blood pressure remained within the normal limits.

Decrease of myocardial deformation parameters, assessed by speckle-tracking echocardiography, precedes LVEF reduction, and may persist during and after chemotherapy^2^. Our research group^8^, similarly with others^10,17^, identified an early decrease of 2D LS or CS, before reduction of LVEF, in patients with cancers treated with anthracyclines. Although still not used routinely, 3D myocardial deformation had demonstrated its usefulness, feasibility, and superiority versus 2D, with good agreement with CMR strain, through independence from geometry-related deformation, direction, and angle^18^. We showed a significant decrease of all 3D deformation parameters after third cycle of CHOP, before 3D LVEF reduction by criteria used for diagnosis of cardiotoxicity. Similarly, Mornos et al.^19^ and Armstrong et al.^20^ showed reduction of 3D LS, RS, or CS after anthracyclines treatment, associated with increased troponin levels, occurring before decrease of LVEF. Current guidelines define occurrence of cardiac dysfunction during chemotherapy by a reduction of global longitudinal strain with more than 15%^2^. We also identified longitudinal strain as a powerful independent predictor for LVEF decrease. Reduction of this parameter by more than 19% after third cycle of CHOP therapy was able to identify patients with chemotherapy-induced cardiotoxicity at the end of treatment with a c-statistics (AUC) of 92%.

Besides cardiac effects, chemotherapy determines negative arterial remodeling with increased arterial stiffness, favoring LV hypertrophy and altered ventricular-arterial coupling, worsening preexisting cardiac dysfunction^21^. In patients with solid or hematological tumors, anthracyclines and adjuvant agents, including CHOP therapy, favor occurrence of arterial stiffness, assessed by increased PWV or WI, persistent after treatment completion^6,22,23^. Narayan et al. demonstrated that early decrease of myocardial deformation and increased ventriculo-arterial coupling are best independent predictors for LVEF reduction^23^. These results are similar to our data, by which we demonstrated an early, progressive, and significant increase of arterial stiffness and altered ventriculo-arterial coupling during chemotherapy, with a significant correlation between vascular function and occurrence of cardiotoxicity, suggesting a possible additional mechanism of chemotherapy-induced cardiac dysfunction. We identified PWV and LS as the best independent predictors for 3D LVEF decrease at the end of chemotherapy. An increase of PWV by more than 27% after the third cycle of CHOP therapy may identify patients with cardiovascular toxicity after chemotherapy with c-statistics (AUC) of 90%. Furthermore, the combination of LS decrease by more than 19% and PWV increase by more than 27% after third cycle of CHOP had a greater prediction value, with a c-statistics (AUC) of 96%.

Cardiac troponin I is the most sensitive and specific biomarker, used for diagnosis of myocardial injury after chemotherapy. On the contrary, NT-pro-BNP, marker of high filling pressures, has a controversial role in early detection of cardiac dysfunction^2^. Several studies demonstrated the role of troponin I and NT-pro-BNP in assessing cardiac dysfunction in hematological neoplasia treated with anthracyclines^7^, however, other studies failed to show contribution of these biomarkers for early detection of cardiotoxicity^24^. In our study, troponin I increased early, while 3D LVEF maintained within normal ranges, persistent after CHOP completion treatment, and significantly higher in group who developed cardiotoxicity. Even if NT-pro-BNP level increased numerically during treatment, no significant difference was found between groups.

Cardiac assessment of cancer patients should be done at baseline, before starting treatment, during chemotherapy and periodically after its completion. In our study, an early cardiac comprehensive evaluation, after only 3 cycles of CHOP (full treatment had 8 ± 2 CHOP cycles) identified predictive parameters for further cardiotoxicity after chemotherapy ended. Follow-up visits are mandatory, in order to diagnose late chemotherapy-induced cardiovascular toxicity.

### Study limitations

First limitation of our study is related to the low number of patients which developed cardiotoxicity (18 out of 110). Comparing with other studies, enrolling between 50 and 70 patients, we enrolled a higher number of oncological patients. However, percentage of those who developed cardiotoxicity was similar in all studies, less than 20%^8,11,14,25^. Second limitation is that we used only proper echo images, without stitching artifacts or poor quality, taking into consideration that quality of ultrasound images can decrease the accuracy of assessment of 3D LV EF and myocardial deformation parameters. However, feasibility of our data was more than 90% for all myocardial segments. Moreover, we used only troponin I, which is currently provided by our laboratory. High-sensitivity troponin I might have a better accuracy, but is not used routinely for the diagnosis of cardiotoxicity. Finally, follow-up of patients after chemotherapy ended is mandatory, knowing the risk of anthracyclines-induced late cardiotoxicity.

## Conclusions

Assessment of 3D longitudinal strain and pulse wave velocity is able to detect early chemotherapy-induced cardiotoxicity, and to predict with good accuracy further decline of 3D LVEF in patients with non-Hodgkin’s lymphoma. Thus, these parameters should be incorporated in clinical protocols, for better monitoring of cardiac function during chemotherapy, and for early intervention.

## Methods

### Study groups

147 consecutive patients diagnosed with NHL, were prospectively enrolled from a single hematology department between January 2014 and October 2018. Inclusion criteria were: age > 18 years; signed informed consent; 3D LVEF > 50%; sinus rhythm; NHL scheduled to receive CHOP chemotherapy according to hematological guidelines. Exclusion criteria were: history of cardiovascular disease; history of radiotherapy. Study protocol was approved by the local ethics committee and conforms with the principles outlined in the Declaration of Helsinki. All patients gave written inform consent to participate in the study. Of the 147 patients, 8 died during chemotherapy due to aggressive forms of NHL, 11 were lost to follow-up and 18 were excluded because of poor image quality on 3DE. Thus, 110 patients (58 ± 11, 51 men) remained in the study.

CHOP chemotherapy consisted of intravenously administration of cyclophosphamide 750 mg/m^2^, vincristine 1.4 mg/m^2^, and doxorubicin 50 mg/m^2^ on day 1, and orally administration of prednisone 100 mg on days 1–5. Full treatment included 8 ± 2 CHOP cycles, repeated at every 21 ± 3 days. Blood collection, 3DE, and echo-tracking were performed at baseline, one day after completion of third cycle, and at end of CHOP chemotherapy. Cardiotoxicity was defined as 3D LVEF reduction below 50%, with more than 10 percentage points, at any time after initiation of CHOP therapy. According to 3D LVEF value at the end of therapy, two groups were defined: patients who developed cardiotoxicity (group I) and patients who did not fulfill these criteria (group II).

### Echocardiography

All ultrasound examinations were performed with a commercially available system equipped with a 4 V probe for 3DE (Vivid E9 Dimension, GE Medical Systems, Horten, Norway). Before every recording, blood pressure and heart rate were measured; one-lead electrocardiogram was used during examination. Images were obtained in left lateral decubitus position, during breath holding, performed by the same investigator and taken in accordance with our previously reported protocol and current guidelines^16,26^. Three cardiac cycles were achieved at each recording. Digital achieved data were analyzed offline using a dedicated software package (EchoPac version BT 12 for PC; GE Medical Systems) with 4D auto-LVQ system. 3DE full-volume LV data sets were achieved in six consecutive electrocardiographically gated subvolumes, with good visualization of all segments, excluding any stitching artifacts. Endocardial border tracing was initiated manually, by identifying two points in 4-chamber view (middle of mitral annulus and LV apex) at end-diastole and end-systole. Then, the software generated semiautomated complete endocardial contour that included papillary muscles and LV outflow tract for end-diastolic and end-systolic volumes^27^; manual edits were performed if needed. 3D strain analysis was the last step of the 4D auto LVQ software. Strain region of interest was automatically generated at end-systole, and had two borders: endocardial, the same used for 3D volumes assessment, and epicardial, generated in previous stage; both contours were adjusted, if necessary. Deformation parameters (longitudinal strain LS, circumferential strain CS, radial strain RS, and area strain AS) were automatically calculated for each of 17 LV segments after tracking was confirmed visually^8^.

### Arterial stiffness

**Figure 3:**
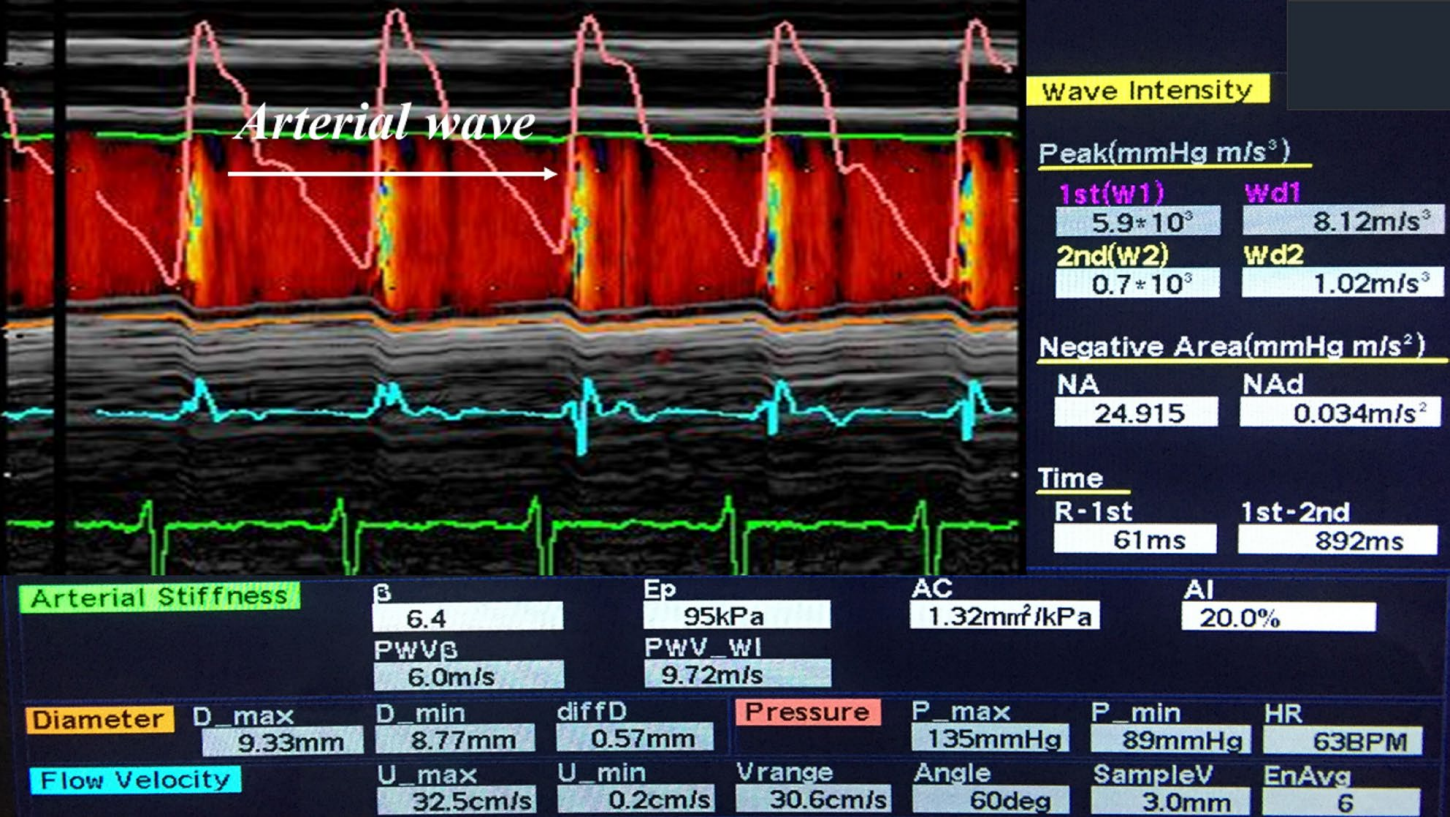
Echo-tracking principle used to measure pulse wave velocity (PWV), augmentation index (AIX), β index, Young modulus of stiffness (Ep), arterial compliance (AC), and Wave Intensity (WI) at the right common carotid artery level, based on the arterial wave obtained from the differences between the arterial diameter in systole and diastole and specific formulas. Modified from^21^.

Echo-tracking system (Aloka Prosound α10, Tokyo, Japan) was used to determine arterial stiffness at right common carotid artery, using our protocol published previously^21,28^. Following parameters of arterial stiffness were assessed: pulse wave velocity (PWV), augmentation index (AIX), beta index, Young modulus of stiffness (Ep), arterial compliance (AC), and wave intensity (WI) (Fig. 3) (detailed definitions and measurements are given in the Supplemental Appendix). WI records two positive peaks: first peak (compression wave), occurs in early systole and depends on LV contractility and second peak (expansion wave), occurs at the end of ejection period and is influenced by LV capacity to stop aortic blood flow. Between the two positive peaks is defined a new parameter, negative area, which represents reflections from the cerebral circulation^28^.

### Troponin I and NT-pro-BNP

Blood was collected in EDTA tubes and TnI and NT-pro-BNP concentrations were measured by CLEIA method with Pathfast Synttergy 2005 analyzer (Mitsubishi Kagaku Iatron, Inc, Tokyo, Japan). The reported value for the 99th percentile for TnI was < 0.02 ng/ml, and less than 112 pg/ml for NT-pro-BNP^29^.

### Reproducibility

For 3D LV EF and deformation parameters, intraobserver, interobserver, and test–retest variability (repeatability) were assessed in our laboratory for 20 consecutive patients, by two observers with same experience. For arterial stiffness and ventriculo-arterial coupling, reproducibility in our laboratory has been reported previously^28^.

### Statistical analysis

SPSS version 19.0 (SPSS, Inc, Chicago, Illinois) was used for data analysis. Results are presented as mean ± standard deviation (SD) or as percentages (%). P < 0.05 was considered significant. Differences between variables of two groups were quantified with independent *t*-test (for means) and *χ*^2^ test (for proportions). Comparisons of parameters within each group at baseline, after third cycle, and after CHOP completion, were assessed with one-way analysis of variance (ANOVA); Scheffé test was used for subgroup analysis. Association between two variables and influence of a parameter on LVEF reduction below 50% at the end of chemotherapy were evaluated by univariate Pearson correlation and multiple linear stepwise regression analysis. Receiver operating characteristic (ROC) curves were obtained for parameters that were predictive for occurrence of cardiotoxicity after the last cycle of CHOP treatment. Intraobserver, interobserver, and repeatability were calculated as 2SD/√2 and reported as percentages from mean value and coefficient of variation^30^.

### Ethical approval

Our research was approved by Ethics Committee of the University and Emergency Hospital of Bucharest

## Supplementary information

Supplementary Information.

## Abbreviations

3DE: 3-Dimensional echocardiography
4D autoLVQ: 4-Dimensional auto left ventricular quantification
AC: Arterial compliance
AIX: Augmentation index
ANOVA: One-way analysis of variance
AS: Area strain
CHOP: Cyclophosphamide, hydroxydaunorubicin (doxorubicin), vincristine, prednisone
CLEIA: Chemo-luminescence enzyme immuno-assay
CMR: Cardiac magnetic resonance
CS: Circumferential strain
EDTA: Ethylenediamine tetra-acetic acid
Ep: Young modulus of stiffness
GE: General electrics
LS: Longitudinal strain
LVEDV: Left ventricular end-diastolic volume
LVEF: Left ventricular ejection fraction
LVESV: Left ventricular end-systolic volume
NHL: Non-Hodgkin’s lymphoma
NT pro-BNP: N-terminal pro-brain natriuretic peptide
PWV: Pulse wave velocity
ROC: Receiver operating curve
RS: Radial strain
SD: Standard deviation
Tn I: Troponin I
WI: Wave intensity
