# Supplementary material for: 3D echocardiography, arterial stiffness, and biomarkers in early diagnosis and prediction of CHOP-induced cardiotoxicity in non-Hodgkin’s lymphoma
Source: Sci Rep. 2020 Oct 28;10:18473. doi: 10.1038/s41598-020-75043-3 (PMC7595195; doi:10.1038/s41598-020-75043-3)
Supplement: Supplementary file 1 — Supplementary Information. [file 41598_2020_75043_MOESM1_ESM.pdf]

Suplemental appendix for the article **“3D echocardiography, arterial stiffness, and biomarkers in early diagnosis and prediction of CHOP-induced cardiotoxicity in non-Hodgkin’s lymphoma”**

Authors: **Diana Mihalcea, Maria Florescu, Ramona Bruja, Natalia Patrascu, Ana-Maria Vladareanu, Dragos Vinereanu**

Definitions and measurements of arterial stiffness parameters by echo-tracking system:

- PWV was calculated from the time delay between two adjacent distension waveforms, using the formula PWV= (dP/dU)/ρ, where dP is the difference between the systolic and diastolic blood pressure, dU is the difference between the arterial diastolic and systolic diameter, and ρ the blood density (1050 kg/m^3);
- Augmentation index (AIX) was measured as AIX= dP/PP, where dP is augmentation pressure (difference between the second and the first systolic peak on the arterial trace) and PP is pulse pressure;
- Beta index of stiffness (β index) was calculated according to the formula β = ln(Ps/Pd)/[(Ds–Dd)/Dd], where ln is the natural logarithm, Ps the systolic blood pressure, Pd the diastolic blood pressure, Ds the arterial systolic diameter and Dd the arterial diastolic diameter;
- Ep, the Young modulus of stiffness, was calculated as: Ep= (Ps–Pd)/[(Ds–Dd)/Dd];
- Arterial compliance (AC) was measured from the arterial cross area and blood pressure, based on the formula: AC = π(Ds × Ds – Dd × Dd) / [4 × (Ps – Pd)] (10);
- Wave intensity (WI), marker of ventriculo-arterial interaction, represents a hemodynamic parameter, measured in real time, by simultaneous recording of RCCA diameter and flow velocity, through the use of a double beam ultrasound system. WI is calculated by the formula WI = (dP/dt)(dU/dt), where P represents blood pressure, U flow velocity by Doppler technique, in respect to time (t).
